# Impact of the Alberta Stroke Program CT Score subregions on long-term functional outcomes in acute ischemic stroke: Results from two multicenter studies in China

**DOI:** 10.2478/jtim-2022-0057

**Published:** 2022-11-15

**Authors:** Xinrui Wang, Caohui Duan, Jinhao Lyu, Dongshan Han, Kun Cheng, Zhihua Meng, Xiaoyan Wu, Wen Chen, Guohua Wang, Qingliang Niu, Xin Li, Yitong Bian, Dan Han, Weiting Guo, Shuai Yang, Ximing Wang, Tijiang Zhang, Junying Bi, Feiyun Wu, Shuang Xia, Dan Tong, Kai Duan, Zhi Li, Rongpin Wang, Jinan Wang, Xin Lou

**Affiliations:** Department of Radiology, Chinese PLA General Hospital, Beijing 100853, China; Department of Radiology, Yuebei People’s Hospital, Shaoguan 512000, Guangdong Province, China; Department of Radiology, Anshan Changda Hospital, Anshan 114000, Liaoning Province, China; Department of Radiology, Shiyan Taihe Hospital, Shiyan 442000, Hubei Province, China; Department of Radiology, Qingdao Municipal Hospital, Qingdao University, Qingdao 266011, Shandong Province, China; Department of Radiology, WeiFang Traditional Chinese Hospital, Weifang 261053, Shandong Province, China; Department of Radiology, The Second Hospital of Jilin University, Jilin University, Changchun 130014, Jilin Province, China; Department of Radiology, The First Affiliated Hospital of Xi’an Jiaotong University, Xi’an 710061, Shaanxi Province, China; Department of Radiology, The First Affiliated Hospital of Kunming Medical University, Kunming Medical University, Kunming 650032, Yunnan Province, China; Department of Radiology, Shanxi Provincial People’s Hospital, Taiyuan 030012, Shanxi Province, China; Department of Radiology, Xiangya Hospital, Central South University, Changsha 410008, Hunan Province, China; Department of Radiology, The First Affiliated Hospital of Soochow University, Soochow University, Suzhou 215006, Jiangsu Province, China; Department of Radiology, The Affiliated Hospital of Zunyi Medical University, Zunyi Medical University, Zunyi 563000, Guizhou Province, China; Department of Radiology, The Third People’s Hospital of Hubei Province, Wuhan 430030, Hubei Province, China; Department of Radiology, The First Affiliated Hospital of Nanjing Medical University, Nanjing Medical University, Nanjing 210029, Jiangsu Province, China; Department of Radiology, Tianjin First Central Hospital, Nankai University, Tianjin 300190, China; Department of Radiology, The First Hospital of Jilin University, Jilin University, Changchun 130021, Jilin Province, China; Department of Radiology, Liangxiang Hospital, Beijing 102401, China; Department of Radiology, The First People’s Hospital of Yunnan Province, Kunming 650034, Yunnan Province, China; Department of Radiology, Guizhou Provincial People’s Hospital, Guiyang 550499, Guizhou Province, China; Department of Radiology, Zhongshan Hospital, Xiamen University, Xiamen 361004, Fujian Province, China

**Keywords:** ischemic stroke, diffusion magnetic resonance imaging, patient outcome assessment

## Abstract

**Background and Objectives:**

The Alberta Stroke Program CT Score (ASPECTS) is a widely used rating system for assessing infarct extent and location. We aimed to investigate the prognostic value of ASPECTS subregions’ involvement in the long-term functional outcomes of acute ischemic stroke (AIS).

**Materials and Methods:**

Consecutive patients with AIS and anterior circulation large-vessel stenosis and occlusion between January 2019 and December 2020 were included. The ASPECTS score and subregion involvement for each patient was assessed using posttreatment magnetic resonance diffusion-weighted imaging. Univariate and multivariable regression analyses were conducted to identify subregions related to 3-month poor functional outcome (modified Rankin Scale scores, 3–6) in the reperfusion and medical therapy cohorts, respectively. In addition, prognostic efficiency between the region-based ASPECTS and ASPECTS score methods were compared using receiver operating characteristic curves and DeLong’s test.

**Results:**

A total of 365 patients (median age, 64 years; 70% men) were included, of whom 169 had poor outcomes. In the reperfusion therapy cohort, multivariable regression analyses revealed that the involvement of the left M4 cortical region in left-hemisphere stroke (adjusted odds ratio [aOR] 5.39, 95% confidence interval [CI] 1.53–19.02) and the involvement of the right M3 cortical region in right-hemisphere stroke (aOR 4.21, 95% CI 1.05–16.78) were independently associated with poor functional outcomes. In the medical therapy cohort, left-hemisphere stroke with left M5 cortical region (aOR 2.87, 95% CI 1.08–7.59) and caudate nucleus (aOR 3.14, 95% CI 1.00–9.85) involved and right-hemisphere stroke with right M3 cortical region (aOR 4.15, 95% CI 1.29–8.18) and internal capsule (aOR 3.94, 95% CI 1.22–12.78) affected were related to the increased risks of poststroke disability. In addition, region-based ASPECTS significantly improved the prognostic efficiency compared with the conventional ASPECTS score method.

**Conclusion:**

The involvement of specific ASPECTS subregions depending on the affected hemisphere was associated with worse functional outcomes 3 months after stroke, and the critical subregion distribution varied by clinical management. Therefore, region-based ASPECTS could provide additional value in guiding individual decision making and neurological recovery in patients with AIS.

## Introduction

The Alberta Stroke Program Early CT Score (ASPECTS) is a semiquantitative scoring system used to estimate the extent of early ischemic changes and to predict clinical outcomes in acute ischemic stroke (AIS).^[1]^ Recent advances have validated the effectiveness of reperfusion therapy, especially endovascular approaches administered in selected patients with large vessel occlusion when treatment is initiated in an appropriate time window.^[2]^ Neuroimaging plays a key role in assessing patients’ eligibility for endovascular therapy.^[3]^ Most landmark randomized clinical trials established their selection criteria, including using ASPECTS as a surrogate of lesion size, where an ASPECTS score of 6 was the cutoff point for treatment.^[4, 5, 6, 7]^

The ASPECTS template divides the middle cerebral artery (MCA) territory into 10 individual regions, with a lower score indicating a larger ischemic area and less potential benefit from revascularization. The total ASPECTS score has been shown to correlate with long-term functional outcomes at the group level; however, the regional effects captured by the ASPECTS have been underestimated. Individual outcomes might be disparate with different hemispheres and locations affected, even when ASPECTS scores are the same. Hemisphere involvement manifests differently from onset symptoms to poststroke recoveries, such as aphasia for the left hemisphere stroke and neglect of the right hemisphere owing to the anatomical asymmetry linked to different brain functions and behaviors.^[8, 9, 10]^ Moreover, infarction of specific regions, such as the motor area, usually increases the risk of poststroke disability, presenting worse functional outcomes quantified by 3-month modified Rankin Scale (mRS) scores. Therefore, the overall ability to solely use the ASPECTS score to discriminate individual outcomes is less reliable, especially when the score is moderate to high (*i.e*., ASPECTS score > 3).^[11]^

Previous studies concerning ASPECTS methodologies have investigated the unequal impact of ASPECTS subregions on functional outcomes. Deep regions, such as the caudate and internal capsule, and cortical regions, such as M3, M4, M5, and M6, were clinically relevant to long-term outcomes. ^[12, 13, 14, 15]^ These studies had inconsistent findings and were almost only conducted on the reperfusion population. Nearly half of the patients with symptomatic large-vessel stenosis initially received medical treatment because of an unclear onset or mild clinical manifestation.^[16]^ Nevertheless, the prognostic value of the ASPECTS subregions on 3-month functional outcomes in this nonthrombolysis cohort remains unclear. Whether there are differences in the distribution of outcome-related ASPECTS subregions between the reperfusion and nonreperfusion cohorts also require further investigation.

Therefore, in this study, we conducted a comprehensive ASPECTS assessment using posttreatment magnetic resonance (MR) diffusion-weighted imaging (DWI) in patients with AIS and anterior circulation large-vessel stenosis and occlusion. We aimed to (1) determine which ASPECTS subregions were related to long-term functional outcomes, depending on the affected hemisphere, (2) compare the distribution of outcome-related subregions between the reperfusion and nonreperfusion cohorts, and (3) validate whether region-based ASPECTS assessment would improve the prognostic efficiency compared with the conventional ASPECTS score.

## Materials and methods

### Study population

We conducted a post hoc analysis of consecutive patients with AIS in two multicenter prospective observational registries, MR-STARS (NCT02580097) and PROTECT (NCT03670862), from January 2019 to December 2020. Both studies aimed to assess the effects of novel imaging biomarkers or imaging patterns on patient selection and outcome prediction in AIS. The local ethics committee approved this study (S2018-193-01). Furthermore, informed consent was obtained from all patients.

The inclusion criteria were as follows: (1) Patients with AIS and severe stenosis or occlusion of the anterior circulation large vessel, including the internal carotid artery (ICA) and MCA; (2) patients with acute infarct lesions within the MCA territory; (3) patients who underwent posttreatment magnetic resonance imaging (MRI); and (4) patients with complete clinical data at admission and outcome assessment using the mRS score after 3 months. We excluded patients with (1) negative findings on DWI; (2) bilateral infarct lesions; (3) old infarct lesions that may affect neurological functional assessment; and (4) cerebral hemorrhage, tumor, or trauma.

Data on patient demographics and vascular risk factors were collected as follows: Age, sex, hypertension, hyperglycemia, hyperlipidemia, coronary heart disease, atrial fibrillation, smoking, and medication history (antiplatelet and lipid-lowering therapy). Stroke severity was assessed on admission using the National Institutes of Health Stroke Scale (NIHSS). Patients underwent either reperfusion therapy (intravenous thrombolysis, endovascular thrombectomy [EVT], or both) or nonreperfusion medical therapy (antiplatelet agents and anticoagulants), as determined by their physicians under the guidelines for managing AIS.^[7]^ Functional outcome was assessed by the 3-month mRS score via clinical interview or telephone follow-up, measuring the degree of poststroke disability in daily activities. The primary outcome was the poor functional outcome, defined as an mRS score of 3–6 at 3 months.

### Imaging protocol

Posttreatment MRIs were performed 24 h to 72 h after symptom onset when the final infarct extent was reached. MRI examinations were performed using a 3-T MR scanner (Discovery 750, GE Healthcare, USA) with a 32-channel head coil using standardized head protocols, including T2 and T1 weighted imaging, T2 fluid-attenuated inversion recovery, DWI, and time-of-flight magnetic resonance angiography (TOF-MRA). The ASPECTS was assessed on DWI with the following scanning parameters: repetition time (TR) 6800 ms, echo time (TE) 90 ms, slice thickness, 5 mm, intersection gap, 1 mm, field of interest (FOV) 24 cm × 24 cm, matrix 128 × 128, number of excitations 1, *b*-values of 0 and 1000 s/mm^2^, number of slices 20. Apparent diffusion coefficient (ADC) maps were simultaneously generated using a mono-exponential fitting model. Arterial status and occlusion site were assessed on TOF-MRA; the scanning parameters were as follows: TR 8.2 ms, TE 3.2 ms, FOV 24 cm × 24 cm, matrix 320 × 320, slice thickness 1 mm, flip angle 12°.

### Imaging analysis

**Figure 1:**
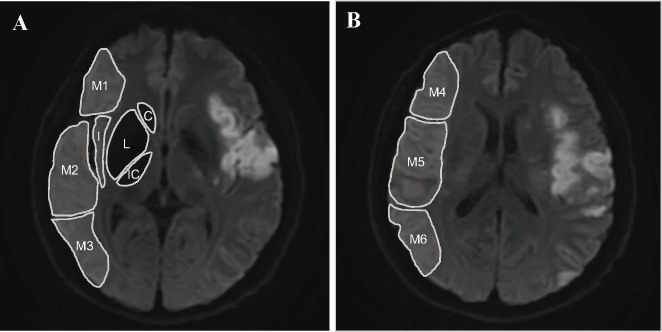
Illustration of the assessment of DWI-ASPECTS. A shows the ganglionic level, which consists of 3 deep subregions (C: caudate nucleus, L: lenticular nucleus, and IC: internal capsule) and 4 cortical subregions (I: insula, M1: anterior inferior frontal lobe, M2: temporal lobe, and M3: inferior parietal and posterior temporal lobe). B shows the supraganglionic level, including 3 cortical subregions superior to M1, M2, and M3 (M4: superior anterior frontal lobe, M5: precentral and superior frontal lobe, and M6: superior parietal lobe). The DWI-ASPECTS score of this patient was rated as 5. DWI-ASPECTS: Alberta Stroke Program Early CT Score on MR-diffusion weighted imaging.

Two neuroradiologists with 5 and 3 years of experience in neuroimaging, blinded to the clinical data, independently performed imaging reviews for each patient, and a third senior neuroradiologist resolved disagreements. Ten subregions of the ASPECTS template were assessed, including three deep regions (caudate nucleus, lenticular nucleus, and internal capsule) and seven cortical regions (insula and M1–M6 regions) at the ganglionic and supraganglionic levels, respectively (Figure 1). Each ASPECTS subregion was scored 0 if totally or partially infarcted or 1 if it appeared normal. Lesions presenting fused hyperintensity on DWI and corresponding ADC value < 620 μm^2^/s were considered abnormal,[^17^] and small hyperintensity spots were excluded. The affected hemisphere and subregions were recorded, and the total DWI-ASPECTS scores were calculated. Arterial status and occlusion site were assessed on TOF-MRA with complete occlusion, defined as a lack of flow signal of a vascular segment and distal vessels, and severe stenosis, defined as severe or critical stenosis of a vascular segment with significant reduction of flow signal distal to the stenosis.^[18]^

### Statistical analysis

Continuous variables were expressed as mean ± standard deviation or median (interquartile range [IQR]). Categorical variables were expressed as counts and percentages. Bivariate comparisons were performed using Student’s *t*-test, the Mann–Whitney *U* test for continuous variables, and the *χ* test (or Fisher’s exact test) for categorical variables. The reliability of ASPECTS assessment between two observers was measured using Cohen κ.

First, univariate analyses were conducted to determine clinical and radiological characteristics related to poor functional outcomes (3-month mRS scores, 3–6) in the reperfusion and medical therapy cohorts. As individual regions were rarely infarcted alone, we calculated Pearson’s correlation coefficient, tolerance, and variance inflation factor (VIF) to detect collinearity among ASPECTS subregions. A correlation coefficient of > 0.7 between two independent variables, tolerance value of < 0.1, or VIF of ≥ 10 for any variable implied that multicollinearity existed.^[19]^ Variables with high collinearity were excluded from the multivariable analyses. Furthermore, we performed two multivariable logistic regression models: an unadjusted model that exclusively contained ASPECTS subregions significant in the univariate analyses and an adjusted model additionally controlling for clinically relevant covariates, such as baseline NIHSS, total ASPECTS score, arterial occlusion sites, and treatment options. Receiver operating characteristic curves were plotted, and DeLong’s test was used to compare the prognostic efficiency of the region-based ASPECTS with the conventional ASPECTS score method. Statistical significance was defined as a two-sided *P*-value of < 0.05. Statistical analyses were performed using the SPSS software version 26 (IBM Corp., NY, USA) and the MedCalc version 19 (MedCalc Software, Mariakerke, Belgium).

## Results

### Study cohort

**Figure 2:**
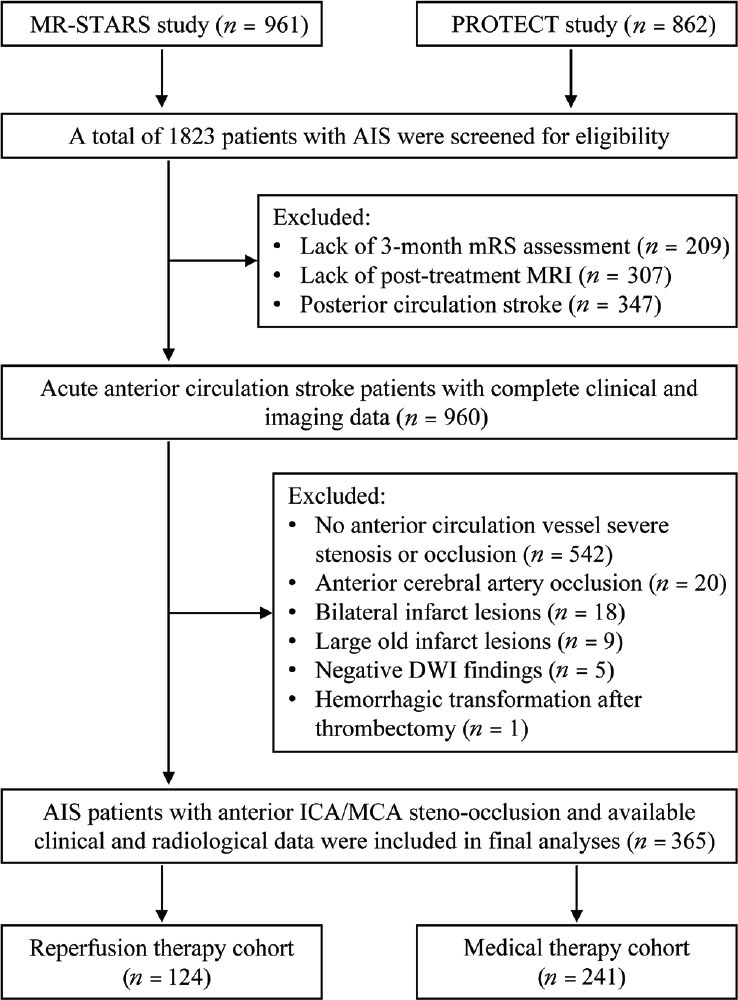
Flowchart of the process of patient inclusion. AIS: acute ischemic stroke; mRS: modified Rankin Scale; DWI: diffusion-weighted imaging; ICA: internal carotid artery; MCA: middle cerebral artery; MRI: magnetic resonance imaging.

Overall, 961 patients from the MR-STARS and 862 from the PROTECT studies were screened for eligibility. A total of 365 patients (median age 64 years [IQR 55–71], 70% men) with available clinical and imaging data were included. Figure 2 shows the flowchart of the patient inclusion process. The baseline clinical characteristics, radiological assessment, and 3-month functional outcomes are summarized in Table 1. A total of 169 (46.3%) patients had poor functional outcomes (3-month mRS scores, 3–6), including 33 deaths during clinical follow-up. The left hemisphere was affected in 203 patients (55.6%), and the right hemisphere was affected in 162 (44.4%).

**Table 1:** Demographics, clinical, and radiological features of the whole cohort

**Characteristics**	**Total (*n* = 365)**
Age, years	64 (55–71)
Male, *n* (%)	257 (70.4)
Hypertension, *n* (%)	230 (63.0)
Hyperglycemia, *n* (%)	113 (31.0)
Hyperlipidemia, *n* (%)	93 (25.5)
Coronary artery disease, *n* (%)	56 (15.3)
Atrial fibrillation, *n* (%)	51 (14.0)
Smoke, *n* (%)	147 (40.3)
Statins, *n* (%)	120 (32.9)
Aspirin, *n* (%)	123 (33.7)
Clopidogrel, *n* (%)	81 (22.2)
Baseline NIHSS	9 (5–12)
Event-to-imaging time, h	7.0 (3.8–14.6)
Reperfusion therapy, *n* (%)	124 (34.0)
Medical therapy, *n* (%)	241 (66.0)
3-month mRS	2 (1–4)
Arterial steno-occlusion site, *n* (%)	
ICA	51 (14.0)
MCA	262 (71.8)
ICA/MCA tandem	52 (14.2)
Left-hemisphere stroke, *n* (%)	203 (55.6)
ASPECTS score	6 (5–8)
Caudate nucleus, *n* (%)	94 (25.8)
Lenticular nucleus, *n* (%)	168 (46.0)
Internal capsule, *n* (%)	92 (25.2)
Insular, *n* (%)	175 (47.9)
M1, *n* (%)	96 (26.3)
M2, *n* (%)	152 (41.6)
M3, *n* (%)	159 (43.6)
M4, *n* (%)	136 (37.3)
M5, *n* (%)	265 (72.6)
M6, *n* (%)	169 (46.3)

NIHSS: National Institute of Health Stroke Scale; mRS: modified Rankin Scale; ICA: internal carotid artery; MCA: middle cerebral artery; ASPECTS: Alberta Stroke Program Early CT Score.

### Characteristics of infarct extent and distribution

The median total DWI-ASPECTS score in our study was 6 (IQR 5–8). Interrater agreement in assessing ASPECTS subregion involvement was excellent, with an overall K coefficient of 0.83 (0.83 and 0.81 for the cortical and deep regions, respectively). The cortical M5 region was the most frequently affected ASPECTS subregion (72.6%), followed by the insula (47.9%) and the M6 region (46.3%). We calculated Pearson’s correlation between individual ASPECTS subregions. The highest correlations were observed between the insula and M2, M1 and M4, and M3 and M6, indicating that these anatomically adjacent regions are more likely to be infarcted simultaneously. No significant collinearity was examined, as the correlation coefficients between the two subregions were < 0.7, the tolerance for each subregion was > 0.1, and all VIF values were < 10. The collinearity diagnostics results are presented in the supplemental material.

### Relationship between ASPECTS subregion involvement and 3-month functional outcome in the reperfusion therapy cohort

Univariate analysis for the relationship between patient characteristics and 3-month poor functional outcome in the reperfusion therapy cohort is shown in Table 2. Compared with patients with functional independence after 3 months, those with poor functional outcomes presented higher baseline NIHSS and lower ASPECTS scores, either in left- or right-sided stroke.

**Table 2:** Univariate analysis for the association of patient characteristics with 3-month poor functional outcome in patients who received reperfusion therapy

Characteristics	Total (*n* = 124)	Left hemisphere			Right hemisphere		
	
		mRS 0–2 (*n* = 40)	mRS 3–6 (*n* = 27)	*P*-value	mRS 0–2 (*n* = 38)	mRS 3–6 (*n* = 19)	*P*-value
Age, years	64 (56–71)	62 (50–68)	68 (59–74)	0.052	66 (56–73)	67 (57–74)	0.939
Male, *n* (%)	88 (71.0)	31 (77.5)	20 (74.1)	0.747	28 (73.7)	9 (47.4)	0.050
Hypertension, *n* (%)	74 (59.7)	22 (55.0)	17 (63.0)	0.517	22 (57.9)	13 (68.4)	0.442
Hyperglycemia, *n* (%)	39 (31.5)	11 (27.5)	7 (25.9)	0.887	13 (34.2)	8 (42.1)	0.560
Hyperlipidemia, *n* (%)	43 (34.7)	19 (47.5)	7 (25.9)	0.075	14 (36.8)	3 (15.8)	0.101
Coronary artery disease, *n* (%)	15 (12.1)	5 (12.5)	3 (11.1)	0.999	6 (15.8)	1 (5.3)	0.405
Atrial fibrillation, *n* (%)	11 (8.9)	2 (5.0)	1 (3.7)	0.999	5 (13.2)	3 (15.8)	0.999
Smoke, *n* (%)	52 (41.9)	18 (45.0)	13 (48.1)	0.800	17 (44.7)	4 (21.1)	0.081
Statins, *n* (%)	49 (39.5)	15 (37.5)	14 (51.9)	0.245	12 (31.6)	8 (42.1)	0.432
Aspirin, *n* (%)	44 (35.5)	14 (35.0)	12 (44.4)	0.436	11 (28.9)	7 (36.8)	0.546
Clopidogrel, *n* (%)	29 (23.4)	8 (20.0)	8 (29.6)	0.365	9 (23.7)	4 (21.1)	0.999
Baseline NIHSS	11 (8–14)	10 (7–12)	14 (9–18)	**0.015**	10 (7–11)	12 (10–15)	**0.011**
Arterial steno-occlusion site, *n* (%)				0.362			0.175
ICA	24 (19.4)	9 (22.5)	3 (11.1)		9 (23.7)	3 (15.8)	
MCA	89 (71.8)	29 (72.5)	21 (77.8)		27 (71.1)	12 (63.2)	
ICA/MCA tandem	11 (8.9)	2 (5.0)	3 (11.1)		2 (5.3)	4 (21.1)	
Treatment option				0.879			0.115
IVT	42 (33.9)	14 (35.0)	9 (33.3)		10 (26.3)	9 (47.4)	
EVT	70 (56.5)	23 (57.5)	15 (55.6)		25 (65.8)	7 (36.8)	
Combined	12 (9.7)	3 (7.5)	3 (11.1)		3 (7.9)	3 (15.8)	
ASPECTS score	6 (4–7)	7 (5–8)	5 (3–7)	**0.017**	6 (5–8)	4 (3–6)	**0.005**
Caudate nucleus, *n* (%)	47 (37.9)	12 (30.0)	15 (55.6)	**0.036**	13 (34.2)	7 (36.8)	0.844
Lenticular nucleus, *n* (%)	70 (56.5)	18 (45.0)	13 (48.1)	0.800	27 (71.1)	12 (63.2)	0.546
Internal capsule, *n* (%)	41 (33.1)	15 (37.5)	10 (37.0)	0.969	7 (18.4)	9 (47.4)	**0.022**
Insula, *n* (%)	64 (51.6)	16 (40.0)	18 (66.7)	**0.032**	16 (42.1)	14 (73.7)	**0.024**
M1, *n* (%)	31 (25.0)	5 (12.5)	9 (33.3)	**0.040**	10 (26.3)	7 (36.8)	0.413
M2, *n* (%)	54 (43.5)	13 (32.5)	16 (59.3)	**0.030**	13 (34.2)	12 (63.2)	**0.038**
M3, *n* (%)	48 (38.7)	16 (40.0)	11 (40.7)	0.952	10 (26.3)	11 (57.9)	**0.020**
M4, *n* (%)	48 (38.7)	6 (15.0)	13 (48.1)	**0.003**	18 (47.4)	11 (57.9)	0.454
M5, *n* (%)	95 (76.6)	28 (70.0)	19 (70.4)	0.974	31 (81.6)	17 (89.5)	0.441
M6, *n* (%)	59 (47.6)	14 (35.0)	17 (63.0)	**0.024**	15 (39.5)	13 (68.4)	**0.039**

NIHSS: National Institute of Health Stroke Scale; mRS: modified Rankin Scale; ICA: internal carotid artery; MCA: middle cerebral artery; IVT: intravenous thrombolysis; EVT: endovascular thrombectomy; ASPECTS: Alberta Stroke Program Early CT Score. The bold fonts indicate statistical significance (*P* < 0.05).

In left-sided stroke, the caudate nucleus, insula, and cortical M1, M2, M4, and M6 regions were more frequently involved in patients with poor clinical outcomes. As there was no collinearity among individual subregions, they were computed as independent variables in the multivariable logistic regression. Only the M4 cortical region involvement (odds ratio [OR] 3.75, 95% confidence interval [CI] 1.10–12.74; *P* = 0.034) was independently associated with unfavorable functional outcomes. After adjusting for clinically relevant variables, the M4 region involvement (adjusted OR [aOR] 5.39, 95% CI 1.53–19.02, *P* = 0.009) remained an independent risk factor for poor clinical outcomes in patients with left-sided stroke.

When stroke lesions involved the right hemisphere, the internal capsule, insula, and cortical M2, M3, and M6 regions were statistically significant in the univariate analysis. Multivariable regression analysis revealed that the M3 cortical region infarction (OR 3.64, 95% CI 1.04–12.82; *P* = 0.044) significantly affected the 3-month poststroke outcome in right-hemisphere stroke. By controlling for clinical risk factors, the adjusted model remained unchanged with the aOR of 4.21 (95% CI 1.05–16.78; *P* = 0.013). The results of the multivariate regression analysis are shown in Table 3.

**Table 3:** Multivariable binary logistic regression analysis for the Alberta stroke program early CT score subregions related to 3-month poor functional outcome

	Unadjusted OR	95% CI	*P*-value	Adjusted OR	95% CI	*P*-value
Reperfusion therapy cohort						
Left - M4	3.75	1.10–12.74	**0.034**	5.39	1.53–19.02	**0.009**
Right - M3	3.64	1.04–12.82	**0.044**	4.21	1.05–16.78	**0.013**
Medical therapy cohort						
Left - Caudate nucleus	5.27	1.76–15.80	**0.003**	3.14	1.00–9.85	**0.049**
Left - M5	2.88	1.14–7.27	**0.025**	2.87	1.08–7.59	**0.034**
Right - Internal capsule	4.83	1.59–14.67	**0.006**	3.94	1.22–12.78	**0.022**
Right - M3	3.16	1.33–7.53	**0.009**	4.15	1.29–8.18	**0.013**

For the reperfusion therapy cohort, the adjusted model additionally adjusted for age, sex, baseline NIHSS, total ASPECTS score, arterial occlusion sites, and treatment options. For the medical therapy cohort, the adjusted model additionally adjusted for baseline NIHSS, total ASPECTS score, and arterial occlusion sites. OR: odds ratio; CI: confidence interval; NIHSS: National Institutes of Health Stroke Scale; ASPECTS: Alberta Stroke Program CT Score. The bold fonts indicate statistical significance (*P* < 0.05).

### Relationship between ASPECTS subregion involvement and 3-month functional outcome in the medical therapy cohort

Univariate analysis of factors affecting the 3-month clinical outcomes in patients who received medical therapy is shown in Table 4. In the univariate analyses, left-hemisphere stroke with left caudate nucleus, insula, and M1–M5 cortical regions affected, or right-hemisphere stroke with right internal capsule, insula, M1–M3, M5, and M6 regions involvement were more prone to worse clinical outcomes. Multivariate regression analysis indicated that the left caudate nucleus (OR 5.27, 95% CI 1.76–15.80, *P* = 0.003) and M5 (OR 2.88, 95% CI 1.14–7.27, *P* = 0.025) cortical region were retained as independent variables relevant to the 3-month functional outcome in left-sided strokes, and the right internal capsule (OR 4.83, 95% CI 1.59–14.67, *P* = 0.006) and M3 (OR 3.16, 95% CI 1.33–7.53, *P* = 0.009) region involvement significantly increased the risk of functional outcome deterioration in right-sided strokes.

**Table 4:** Univariate analysis for the association of patient characteristics with 3-month poor functional outcome in patients who received medical therapy

Characteristics	Total (*n* = 241)	Left hemisphere			Right hemisphere		
	
		mRS 0–2 (*n* = 70)	mRS 3–6 (*n* = 66)	*P*-value	mRS 0–2 (*n* = 48)	mRS 3–6 (*n* = 57)	*P*-value
Age, years	65 (55–70)	62 (53–71)	65 (54–70)	0.505	64 (53–70)	66 (60–70)	0.143
Male, *n* (%)	169 (70.1)	50 (71.4)	49 (74.2)	0.712	35 (72.9)	35 (61.4)	0.213
Hypertension, *n* (%)	156 (64.7)	48 (68.6)	43 (65.2)	0.672	28 (58.3)	37 (64.9)	0.489
Hyperglycemia, *n* (%)	74 (30.7)	26 (37.1)	18 (27.3)	0.219	15 (31.3)	15 (26.3)	0.577
Hyperlipidemia, *n* (%)	50 (20.7)	15 (21.4)	10 (15.2)	0.345	9 (18.8)	16 (28.1)	0.264
Coronary artery disease, *n* (%)	41 (17.0)	14 (20.0)	11 (16.7)	0.616	7 (14.6)	9 (15.8)	0.864
Atrial fibrillation, *n* (%)	40 (16.6)	14 (20.0)	10 (15.2)	0.459	7 (14.6)	9 (15.8)	0.864
Smoke, *n* (%)	95 (39.4)	30 (42.9)	25 (37.9)	0.554	16 (33.3)	24 (42.1)	0.356
Statins, *n* (%)	71 (29.5)	19 (27.1)	21 (31.8)	0.550	14 (29.2)	17 (29.8)	0.941
Aspirin, *n* (%)	79 (32.8)	22 (31.4)	21 (31.8)	0.961	19 (39.6)	17 (29.8)	0.294
Clopidogrel, *n* (%)	52 (21.6)	15 (21.4)	14 (21.2)	0.975	13 (27.1)	10 (17.5)	0.239
Baseline NIHSS	8 (4–11)	6 (2–9)	11 (7–15)	**<0.001**	5 (3–8)	9 (6–12)	**<0.001**
Arterial steno-occlusion site, *n* (%)				0.073			0.244
ICA	27 (11.2)	7 (10.0)	8 (12.1)		3 (6.3)	9 (15.8)	
MCA	173 (71.8)	55 (78.6)	41 (62.1)		36 (75.0)	41 (71.9)	
ICA/MCA tandem	41 (17.0)	8 (11.4)	17 (25.8)		9 (18.8)	7 (12.3)	
ASPECTS score	6 (5–8)	7 (6–8)	6 (4–7)	**<0.001**	7 (5–8)	5 (3–7)	**<0.001**
Caudate nucleus, *n* (%)	47 (19.5)	7 (10.0)	21 (31.8)	**0.002**	7 (14.6)	12 (21.1)	0.391
Lenticular nucleus, *n* (%)	98 (40.7)	22 (31.4)	27 (40.9)	0.250	20 (41.7)	29 (50.9)	0.346
Internal capsule, *n* (%)	51 (21.2)	12 (17.1)	14 (21.2)	0.546	6 (12.5)	19 (33.3)	**0.013**
Insula, *n* (%)	111 (46.1)	23 (32.9)	39 (59.1)	**0.002**	17 (35.4)	32 (56.1)	**0.034**
M1, *n* (%)	65 (27.0)	12 (17.1)	23 (34.8)	**0.018**	9 (18.8)	21 (36.8)	**0.041**
M2, *n* (%)	98 (40.7)	19 (27.1)	35 (53.0)	**0.002**	15 (31.3)	29 (50.9)	**0.042**
M3, *n* (%)	111 (46.1)	23 (32.9)	33 (50.0)	**0.042**	19 (39.6)	36 (63.2)	**0.016**
M4, *n* (%)	88 (36.5)	16 (22.9)	33 (50.0)	**0.001**	16 (33.3)	23 (40.4)	0.458
M5, *n* (%)	170 (70.5)	40 (57.1)	54 (81.8)	**0.002**	30 (62.5)	46 (80.7)	**0.038**
M6, *n* (%)	110 (45.6)	28 (40.0)	37 (56.1)	0.061	15 (31.3)	30 (52.6)	**0.027**

NIHSS: National Institute of Health Stroke Scale; mRS: modified Rankin Scale; ICA: internal carotid artery; MCA: middle cerebral artery; ASPECTS: Alberta Stroke Program Early CT Score. The bold fonts indicate statistical significance (*P* < 0.05).

After adjusting for the baseline NIHSS score, arterial steno-occlusion site, and total ASPECTS score, the adjusted models remained unchanged. Left-sided stroke with left caudate nucleus (aOR 3.14, 95% CI 1.00–9.85, *P* = 0.049) and cortical M5 region (aOR 2.87, 95% CI 1.08–7.59, *P* = 0.034) involvement, or right-sided stroke affecting the right internal capsule (aOR 3.94, 95% CI 1.22–12.78, *P* = 0.022) and cortical M3 region (aOR 4.15, 95% CI 1.29–8.18, *P* = 0.013) were significantly associated with poor functional outcomes 3 months after stroke (Table 3).

### Comparison of prognostic efficiency between region-based ASPECTS and ASPECTS score assessment

**Figure 3:**
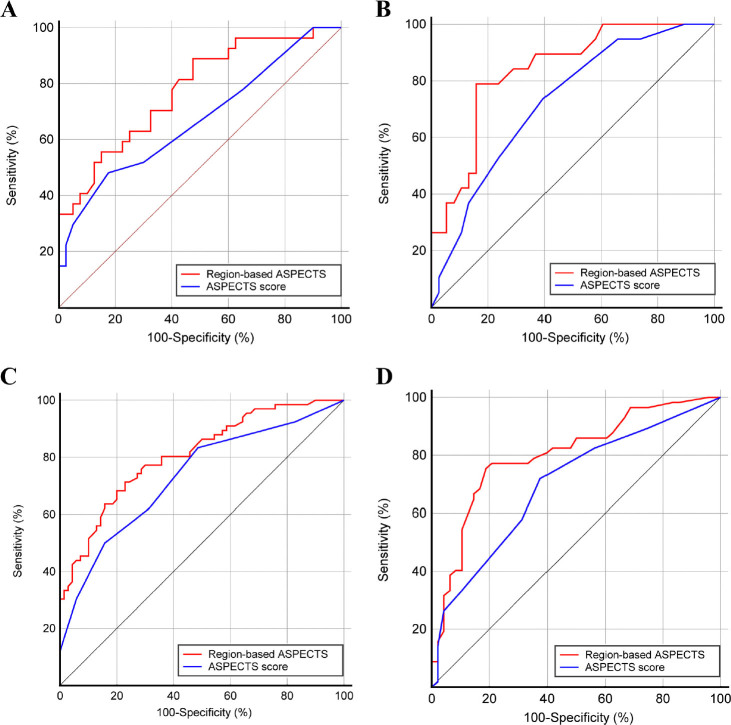
Receiver operating characteristic curves of region-based ASPECTS assessment and ASPECTS score for predicting 3-month poor functional outcome in the reperfusion therapy cohort with left-sided (A) and right-sided (B) strokes and in the medical therapy cohort with left-sided (C) and right-sided (D) stroke, respectively. ASPECTS: Alberta Stroke Program Early CT Score.

We compared the prognostic efficiency of region-based ASPECTS assessment with the conventional ASPECTS score method. The ROC curves are plotted in Figure 3. As shown in Table 5, the area under the ROC curves of the region-based ASPECTS method was significantly higher than the ASPECTS score in the reperfusion and medical therapy cohorts (all DeLong’s test *P-*values < 0.05). In left-sided stroke, the specificity of the region-based ASPECTS method was no better than that of the ASPECTS score; however, the sensitivity for identifying patients with unfavorable poststroke functional outcomes was notably improved. In right-sided stroke, the sensitivity and specificity were higher using the region-based ASPECTS assessment than the conventional ASPECTS score method.

**Table 5:** Prognostic performance of region-based ASPECTS assessment and ASPECTS score method

	AUC	95% CI	Sensitivity (%)	Specificity (%)
Reperfusion therapy cohort with left hemisphere affected				
Region-based ASPECTS	0.776	0.658–0.869	77.8	72.5
ASPECTS score	0.670	0.545–0.760	51.9	82.5
Reperfusion therapy cohort with right hemisphere affected				
Region-based ASPECTS	0.840	0.719–0.924	78.9	84.2
ASPECTS score	0.726	0.591–0.836	73.7	60.5
Medical therapy cohort with left hemisphere affected				
Region-based ASPECTS	0.809	0.733–0.871	71.2	77.1
ASPECTS score	0.731	0.649–0.804	50.0	84.3
Medical therapy cohort with right hemisphere affected				
Region-based ASPECTS	0.803	0.714–0.874	75.4	81.2
ASPECTS score	0.701	0.604–0.787	71.9	62.5

AUC: area under the receiver operating characteristic curve; CI: confidence interval; ASPECTS: Alberta Stroke Program CT Score.

**Figure 4:**
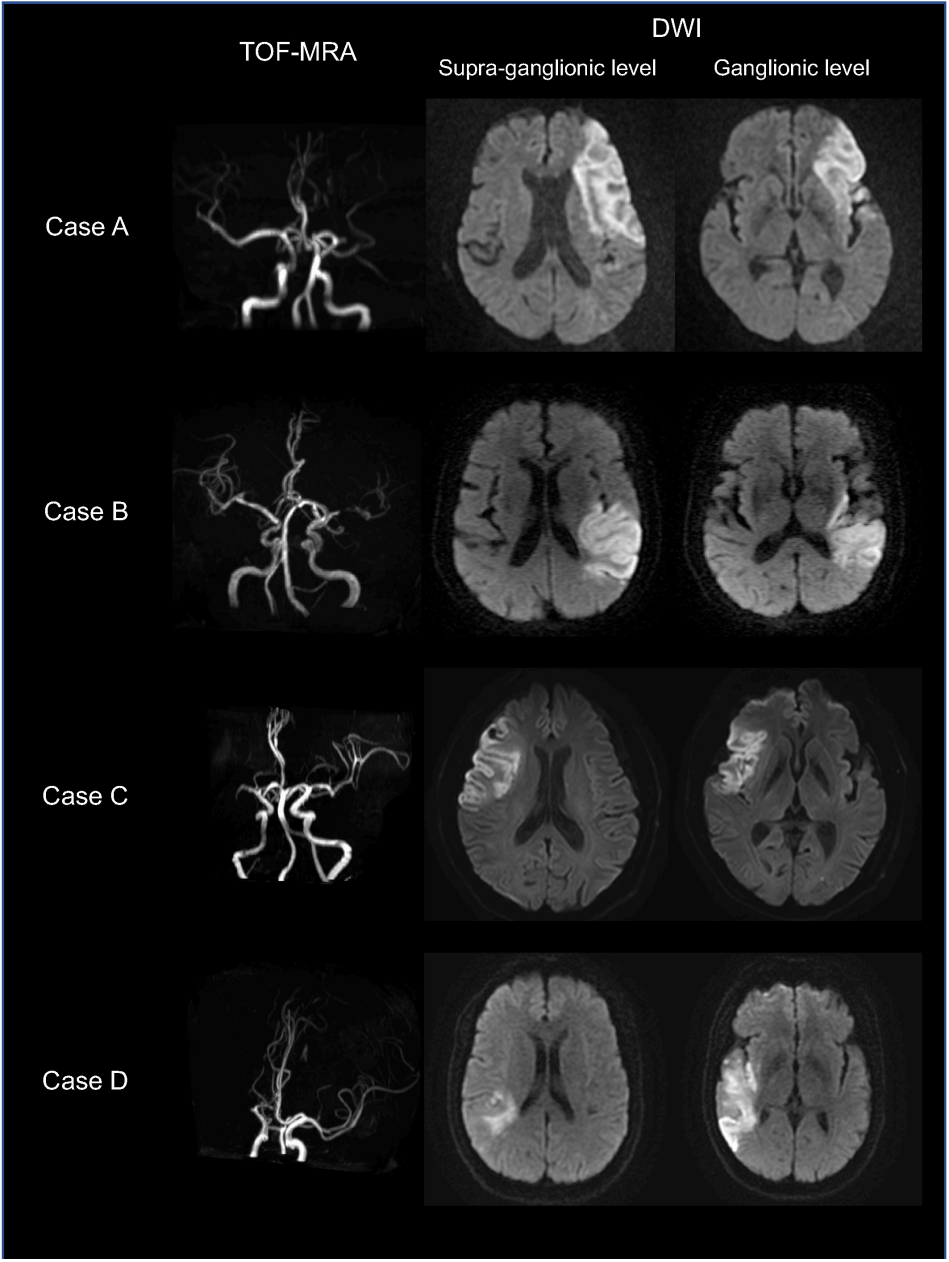
Representative cases of patients with different ASPECTS subregions involvement and clinical outcomes. Case A: A 73-year-old man with left MCA occlusion on TOF-MRA. The baseline NIHSS score was 12. Posttreatment MR-DWI showed the infarct lesion involving the left insula, M1, M4, and M5 regions with an ASPECTS of 6. This patient received intravenous thrombolysis therapy yet suffered moderately severe disability with a 3-month mRS score of 4. Case B: A 73-year-old woman with left MCA severe stenosis and baseline NIHSS score of 9. The infarct lesion affected the left insula, M3, M5, and M6 (ASPECTS score = 6). This patient received medical therapy and presented slight poststroke disability with a 3-month mRS score of 2. Case C: A 69-year-old man with right MCA occlusion and baseline NIHSS score of 8. The infarct lesion involved the right insula, M1, M2, M4, and M5 (ASPECTS score = 5). This patient presented slight poststroke disability with an mRS score of 2 at 3 months. Case D: A 53-year-old man with right MCA occlusion and baseline NIHSS score of 4. Posttreatment DWI showed the infarct lesion involving the right insula, M2, M3, and M6 (ASPECTS score = 6). This patient presented moderate disability with a 3-month mRS score of 3. MCA: middle cerebral artery; TOF-MRA: time-of-flight MR angiography; NIHSS: National Institute of Health Stroke Scale; mRS: modified Rankin Scale; ASPECTS: Alberta Stroke Program Early CT Score; MR: magnetic resonance; DWI: diffusion-weighted imaging.

Figure 4 shows representative cases of patients with similar ASPECTS scores; however, infarct lesions involved different regions and caused different functional outcomes.

## Discussion

Our study observed that the involvement of specific ASPECTS subregions depending on the affected hemisphere was associated with poor functional outcomes 3 months after stroke, and the critical subregion distribution varied by clinical management. In patients receiving reperfusion therapy, left-sided stroke with cortical M4 involvement and right-sided stroke with cortical M3 involvement were independently associated with increased disability. In the medical therapy cohort, ischemic lesions involving the left caudate nucleus and cortical M5 region or the right internal capsule and cortical M3 region were independent risk factors for worse outcomes. The region-based ASPECTS method would be more informative in assessing stroke extent and topography and improving prognostic efficiency compared with the conventional ASPECTS score.

The ASPECTS method was initially designed on noncontrast CT to measure infarcted regions in the anterior circulation as a surrogate of infarct volume and to link acute infarct locations to long-term stroke outcomes. ^[10, 20]^ Our study corresponded with prior studies on the unequal impact of ASPECTS subregions on 3-month clinical outcomes. However, existing studies have only been conducted on reperfusion cohorts using either CT-ASPECTS ^[15, 21]^ or DWI-ASPECTS ^[12, 13, 14]^ and have yielded inconsistent results. Several reasons may explain this discrepancy. First, subtle density changes in early ischemic lesions were hardly captured by CT-ASPECTS. In contrast, DWI-ASPECTS was more sensitive for detecting early ischemia with a higher interrater agreement. ^[22, 23, 24]^ Second, there was a wide imaging time range meaning that pretreatment scans might insufficiently capture the full extent of the final infarct lesions. Moreover, most studies lacked the statistical power to control other confounders potentially associated with poststroke functional outcomes. It remains unclear whether a region-based ASPECTS assessment could improve prognostic efficiency compared with the ASPECTS score.

We conducted a posttreatment DWI-ASPECTS assessment with a relatively limited time interval of 24 h to 72 h after stroke. The interrater agreement was excellent, indicating the reliability of this imaging tool in assessing the acute infarct burden and topography. Our results contribute to clinical literature by describing critical regions relevant to 3-month poor outcomes by controlling for clinical confounders in the reperfusion and nonreperfusion cohorts. In patients who received reperfusion therapy, left-sided stroke involving the M4 region or right-sided stroke involving the M3 region was independently associated with an increased risk of poor functional outcomes. The M4 region represents the superior frontal cortex. Recent studies have indicated that damage to the left superior frontal cortex influences semantic and phonological fluency and interferes with long-term memory, attention, and visuospatial function recovery after stroke.^[25, 26]^ While the initial symptom, such as aphasia after a left-sided stroke, is easily recognized, neglect caused by right-hemisphere stroke is sometimes subtle, causing a delay in treatment initiation and reduction of rehabilitation effects.^[10, 27, 28]^ Infarctions involving the right temporoparietal junction (namely the right M3 region) often cause unilateral spatial neglect due to attention network dysfunction and are associated with worse functional outcomes.^[29, 30]^

In patients who received medical therapy, the left M5 cortical region and deep caudate nucleus involved in left-hemisphere stroke and the right M3 cortical region and internal capsule involved in right-hemisphere stroke were more likely to increase the risk of poststroke disability. In left-hemisphere stroke, the involvement of the frontoparietal cortex (M5 region) is likely to impair the language center. The concomitant injury of subcortical white matter might affect the adjacent pathway of the superior longitudinal fasciculus and impair cognitive function, which in turn influences functional aphasia recovery.^[31, 32, 33]^ The left caudate nucleus is an associative structure linking to the frontal and the parietal lobes, and once infarcted, it might affect motor and cognitive recovery.^[33, 34, 35]^ For right-hemisphere stroke, in addition to cortical M3 region involvement, which was consistent with the reperfusion cohort, internal capsule injury was clinically relevant with worse functional outcomes. The internal capsule is a deep region within the corticospinal tract passing through.^[35, 36]^ Instead of directly affecting motor activity, degeneration of tracts in the right internal capsule might cause other poststroke complications, such as dysphagia, which extends hospitalization and increases mortality.^[37]^

When comparing outcome-related ASPECTS subregions between the reperfusion and medical therapy cohorts, a noteworthy fact was that deep regions (left caudate nucleus and right internal capsule) involvement was an independent risk factor only in the medical therapy cohort. The irreversible infarction of the deep nuclei indicated insufficient deep collateral support due to long-term arterial stenosis, which was also more prone to larger ischemic extents and worse clinical outcomes.

From a clinical standpoint, there is clear evidence that a larger infarct extent, namely a lower ASPECTS score is usually associated with increased disability. Therefore, the goal of this tool is not to be a perfect discriminator of poor functional outcomes but to add objective evidence to help clinical management when different patients present with the same moderate-to-high total ASPECTS score. As shown in Figure 4, critical subregion involvement would be an imaging marker for predicting poor functional outcomes, indicating that more intensive monitoring and clinical intervention are needed to prevent functional deterioration.

This study has some limitations. First, we conducted post hoc analyses of two prospective multicenter registries and identified critical ASPECTS subregions related to long-term functional outcomes in the reperfusion and nonreperfusion therapy cohorts. Our results require further verification in larger sample sizes and external cohorts, and if possible, ascribe modified weights to the conventional ASPECTS scoring system for higher prognostic efficiency. Second, as the brain tissue volume differed among the ASPECTS subregions, the final infarct volumes varied when different subregions were infarcted. We estimated the infarct extent using the total ASPECTS score instead of measuring the actual infarct volume. We strictly assessed each ASPECTS subregion to ensure the accuracy of the evaluation of the infarct extent, as a subregion presenting fused hyperintensity on DWI was identified as abnormal to avoid significant volumetric differences. A previous study has also shown that the region-specific impact on outcomes remained unchanged regardless of the infarct volume.^[13]^ Third, we developed corresponding norms before the start of the two multicenter studies; however, we could not guarantee the complete unification of clinical management and imaging protocols among research institutions. We conducted a comprehensive and careful data screening to ensure that the final patients included in this study met the inclusion criteria.

In conclusion, our study demonstrated that the involvement of specific ASPECTS subregions was associated with worse functional outcomes 3 months after stroke depending on the affected hemisphere, and the critical subregion distribution varied by clinical management. Incorporating ischemic topologic properties captured by the ASPECTS would improve the prognostic efficiency and provide additional benefits in stroke management and neurologic recovery in patients with AIS.

## Supplementary Material

Supplementary Material

## References

[1] Barber PA, Demchuk AM, Zhang J, Buchan AM. (2000). Validity and reliability of a quantitative computed tomography score in predicting outcome of hyperacute stroke before thrombolytic therapy. ASPECTS Study Group. Alberta Stroke Programme Early CT Score. Lancet.

[2] Saver JL, Goyal M, van der Lugt A, Menon BK, Majoie CB, Dippel DW (2016). Time to treatment with endovascular thrombectomy and outcomes from ischemic stroke: a meta-analysis. JAMA.

[3] Menon BK, Campbell BC, Levi C, Goyal M. (2015). Role of imaging in current acute ischemic stroke workflow for endovascular therapy. Stroke.

[4] Goyal M, Demchuk AM, Menon BK, Eesa M, Rempel JL, Thornton J (2015). Randomized assessment of rapid endovascular treatment of ischemic stroke. N Engl J Med.

[5] Jovin TG, Chamorro A, Cobo E, de Miquel MA, Molina CA, Rovira A (2015). Thrombectomy within 8 hours after symptom onset in ischemic stroke. N Engl J Med.

[6] Saver JL, Goyal M, Bonafe A, Diener HC, Levy EI, Pereira VM (2015). Stent-retriever thrombectomy after intravenous t-PA vs. t-PA alone in stroke. N Engl J Med.

[7] Powers WJ, Rabinstein AA, Ackerson T, Adeoye OM, Bambakidis NC, Becker K (2019). Guidelines for the early management of patients with acute ischemic stroke: 2019 update to the 2018 guidelines for the early management of acute ischemic stroke: a guideline for healthcare professionals from the American Heart Association/American Stroke Association. Stroke.

[8] Güntürkün O, Ströckens F, Ocklenburg S. (2020). Brain lateralization: a comparative perspective. Physiol Rev.

[9] Lou Y, Zhao L, Yu S, Sun B, Hou Z, Zhang Z (2020). Brain asymmetry differences between Chinese and Caucasian populations: a surface-based morphometric comparison study. Brain Imaging Behav.

[10] Etherton MR, Rost NS, Wu O. (2018). Infarct topography and functional outcomes. J Cereb Blood Flow Metab.

[11] Weir NU, Pexman JH, Hill MD, Buchan AM. (2006). How well does ASPECTS predict the outcome of acute stroke treated with IV tPA?. Neurology.

[12] Panni P, Michelozzi C, Blanc R, Chen B, Consoli A, Mazighi M (2020). The role of infarct location in patients with DWI-ASPECTS 0-5 acute stroke treated with thrombectomy. Neurology.

[13] Rangaraju S, Streib C, Aghaebrahim A, Jadhav A, Frankel M, Jovin TG. (2015). Relationship between lesion topology and clinical outcome in anterior circulation large vessel occlusions. Stroke.

[14] Rosso C, Blanc R, Ly J, Samson Y, Lehéricy S, Gory B (2019). Impact of infarct location on functional outcome following endovascular therapy for stroke. J Neurol Neurosurg Psychiatry.

[15] Seyedsaadat SM, Neuhaus AA, Nicholson PJ, Polley EC, Hilditch CA, Mihal DC (2021). Differential contribution of ASPECTS regions to clinical outcome after thrombectomy for acute ischemic stroke. AJNR Am J Neuroradiol.

[16] Guisado-Alonso D, Martinez-Domeno A, Prats-Sanchez L, Delgado-Mederos R, Camps-Renom P, Abilleira S (2021). Reasons for not performing mechanical thrombectomy: a population-based study of stroke codes. Stroke.

[17] Albers GW, Lansberg MG, Kemp S, Tsai JP, Lavori P, Christensen S (2017). A multicenter randomized controlled trial of endovascular therapy following imaging evaluation for ischemic stroke (DEFUSE 3). Int J Stroke.

[18] Fiebach JB, Al-Rawi Y, Wintermark M, Furlan AJ, Rowley HA, Lindstén A (2012). Vascular occlusion enables selecting acute ischemic stroke patients for treatment with desmoteplase. Stroke.

[19] Liao Y, Yin G, Fan X. (2020). The positive lymph node ratio predicts survival in T(1-4)N(1-3)M(0) non-small cell lung cancer: a nomogram using the SEER database. Front Oncol.

[20] Pexman JH, Barber PA, Hill MD, Sevick RJ, Demchuk AM, Hudon ME (2001). Use of the Alberta Stroke Program Early CT Score (ASPECTS) for assessing CT scans in patients with acute stroke. AJNR Am J Neuroradiol.

[21] Phan TG, Demchuk A, Srikanth V, Silver B, Patel SC, Barber PA (2013). Proof of concept study: relating infarct location to stroke disability in the NINDS rt-PA trial. Cerebrovasc Dis.

[22] Mitomi M, Kimura K, Aoki J, Iguchi Y. (2014). Comparison of CT and DWI findings in ischemic stroke patients within 3 hours of onset. J Stroke Cerebrovasc Dis.

[23] Payabvash S, Noorbaloochi S, Qureshi AI. (2017). Topographic assessment of acute ischemic changes for prognostication of anterior circulation stroke. J Neuroimaging.

[24] Schröder J, Thomalla G. (2016). A critical review of alberta stroke program early ct score for evaluation of acute stroke imaging. Front Neurol.

[25] Stockert A, Wawrzyniak M, Klingbeil J, Wrede K, Kümmerer D, Hartwigsen G (2020). Dynamics of language reorganization after left temporo-parietal and frontal stroke. Brain.

[26] Schmidt CSM, Nitschke K, Bormann T, Römer P, Kümmerer D, Martin M (2019). Dissociating frontal and temporal correlates of phonological and semantic fluency in a large sample of left hemisphere stroke patients. Neuroimage Clin.

[27] Goto A, Okuda S, Ito S, Matsuoka Y, Ito E, Takahashi A (2009). Locomotion outcome in hemiplegic patients with middle cerebral artery infarction: the difference between right- and left-sided lesions. J Stroke Cerebrovasc Dis.

[28] Ween JE, Alexander MP, D’Esposito M, Roberts M. (1996). Factors predictive of stroke outcome in a rehabilitation setting. Neurology.

[29] Umarova RM, Nitschke K, Kaller CP, Klöppel S, Beume L, Mader I (2016). Predictors and signatures of recovery from neglect in acute stroke. Ann Neurol.

[30] Vallar G, Calzolari E. (2018). Unilateral spatial neglect after posterior parietal damage. Handb Clin Neurol.

[31] Kyeong S, Kang H, Kyeong S, Kim DH. (2019). Differences in brain areas affecting language function after stroke. Stroke.

[32] Marchina S, Zhu LL, Norton A, Zipse L, Wan CY, Schlaug G. (2011). Impairment of speech production predicted by lesion load of the left arcuate fasciculus. Stroke.

[33] Munsch F, Sagnier S, Asselineau J, Bigourdan A, Guttmann CR, Debruxelles S (2016). Stroke location is an independent predictor of cognitive outcome. Stroke.

[34] Ward P, Seri Cavanna AE. (2013). Functional neuroanatomy and behavioural correlates of the basal ganglia: evidence from lesion studies. Behav Neurol.

[35] Payabvash S, Souza LC, Kamalian S, Wang Y, Passanese J, Kamalian S (2012). Location-weighted CTP analysis predicts early motor improvement in stroke: a preliminary study. Neurology.

[36] Rosso C, Valabregue R, Attal Y, Vargas P, Gaudron M, Baronnet F (2013). Contribution of corticospinal tract and functional connectivity in hand motor impairment after stroke. PLoS One.

[37] Yang HE, Kang H, Kyeong S, Kim DH. (2022). Structural connectivity affecting aspiration after stroke. Dysphagia.

