# Supplementary material for: Impact of the Alberta Stroke Program CT Score subregions on long-term functional outcomes in acute ischemic stroke: Results from two multicenter studies in China
Source: J Transl Int Med. 2022 Nov 15;12(2):197–208. doi: 10.2478/jtim-2022-0057 (PMC11107184; doi:10.2478/jtim-2022-0057)
Supplement: Supplementary file 1 — Supplementary Material [file jtim-2022-0057_sm.pdf]

Supplementary Material

Table S1: Pearson's correlation between subregions captured by the ASPECTS in the reperfusion therapy cohort

ASPECTS subregions	C	L	IC	I	M1	M2	M3	M4	M5	M6
C	1	0.452**	0.334**	0.152	0.163	0.85	−0.041	0.164	−0.118	0.021
L		1	0.375**	−0.102	−0.019	−0.114	−0.204*	0.097	−0.063	−0.205*
IC			1	0.132	0.069	−0.030	−0.031	0.040	−0.098	0.017
I				1	0.298**	0.590**	0.173	0.239**	0.151	0.309**
M1					1	0.432**	0.191*	0.459**	0.099	0.196*
M2						1	0.270**	0.204*	0.293**	0.368**
M3							1	0.252**	0.165	0.503**
M4								1	0.322**	0.171
M5									1	0.221*
M6										1

*indicates correlation is significant at the 0.05 level. ** indicates correlation is significant at the 0.01 level. ASPECTS: Alberta Stroke Program Early CT Score; C: caudate nucleus; L: lenticular nucleus; IC: internal capsule; I: insula.

Table S2: Pearson's correlation between subregions captured by the ASPECTS in the medical therapy cohort

ASPECTS subregions	C	L	IC	I	M1	M2	M3	M4	M5	M6
C	1	0.488**	0.335**	0.091	0.149*	0.083	0.007	0.105	0.019	0.012
L		1	0.398**	0.015	0.182**	−0.015	−0.155*	0.056	−0.058	−0.165*
IC			1	0.072	0.074	0.026	0.010	0.050	−0.133*	−0.006
I				1	0.339**	0.659**	0.098	0.198**	0.323**	0.189**
M1					1	0.353**	0.095	0.627**	0.311**	0.100
M2						1	0.235**	0.179**	0.387**	0.259**
M3							1	0.095	0.141*	0.524**
M4								1	0.301**	0.101
M5									1	0.154*
M6										1

*indicates correlation is significant at the 0.05 level. **indicates correlation is significant at the 0.01 level. ASPECTS: Alberta Stroke Program Early CT Score; C: caudate nucleus; L: lenticular nucleus; IC: internal capsule; I: insula.

Table S3: The tolerance and variance inflation factor for the reperfusion and medical therapy cohorts

ASPECTS subregions	Reperfusion cohort		Medical therapy cohort	
	Tolerance	VIF	Tolerance	VIF
C	0.698	1.432	0.723	1.383
L	0.649	1.541	0.640	1.563
IC	0.781	1.280	0.785	1.273
I	0.586	1.701	0.540	1.853
M1	0.642	1.559	0.515	1.942
M2	0.497	2.013	0.493	2.030
M3	0.694	1.441	0.697	1.435
M4	0.639	1.564	0.580	1.723
M5	0.773	1.293	0.760	1.317
M6	0.656	1.523	0.691	1.446

VIF: variance inflation factor; ASPECTS: Alberta Stroke Program Early CT Score; C: caudate nucleus; L: lenticular nucleus; IC: internal capsule; I: insula.
